# In-depth analysis of genomes and functional genomics of orchid using cutting-edge high-throughput sequencing

**DOI:** 10.3389/fpls.2022.1018029

**Published:** 2022-09-23

**Authors:** Cheng Song, Yan Wang, Muhammad Aamir Manzoor, Di Mao, Peipei Wei, Yunpeng Cao, Fucheng Zhu

**Affiliations:** ^1^ College of Biological and Pharmaceutical Engineering, West Anhui University, Lu’an, China; ^2^ School of Life Science, Anhui Agricultural University, Hefei, China; ^3^ Albrecht Daniel Thaer Institute for Agricultural and Horticultural Sciences, Humboldt University of Berlin, Berlin, Germany; ^4^ Chinese Academy of Sciences (CAS) Key Laboratory of Plant Germplasm Enhancement and Specialty Agriculture, Wuhan Botanical Garden, Chinese Academy of Sciences, Wuhan, China

**Keywords:** third-generation sequencing, orchid, genome assembly, polyploidy, functional genomics, molecular breeding

## Abstract

High-throughput sequencing technology has been facilitated the development of new methodologies and approaches for studying the origin and evolution of plant genomes and subgenomes, population domestication, and functional genomics. Orchids have tens of thousands of members in nature. Many of them have promising application potential in the extension and conservation of the ecological chain, the horticultural use of ornamental blossoms, and the utilization of botanical medicines. However, a large-scale gene knockout mutant library and a sophisticated genetic transformation system are still lacking in the improvement of orchid germplasm resources. New gene editing tools, such as the favored CRISPR-Cas9 or some base editors, have not yet been widely applied in orchids. In addition to a large variety of orchid cultivars, the high-precision, high-throughput genome sequencing technology is also required for the mining of trait-related functional genes. Nowadays, the focus of orchid genomics research has been directed to the origin and classification of species, genome evolution and deletion, gene duplication and chromosomal polyploidy, and flower morphogenesis-related regulation. Here, the progressing achieved in orchid molecular biology and genomics over the past few decades have been discussed, including the evolution of genome size and polyploidization. The frequent incorporation of LTR retrotransposons play important role in the expansion and structural variation of the orchid genome. The large-scale gene duplication event of the nuclear genome generated plenty of recently tandem duplicated genes, which drove the evolution and functional divergency of new genes. The evolution and loss of the plastid genome, which mostly affected genes related to photosynthesis and autotrophy, demonstrated that orchids have experienced more separate transitions to heterotrophy than any other terrestrial plant. Moreover, large-scale resequencing provide useful SNP markers for constructing genetic maps, which will facilitate the breeding of novel orchid varieties. The significance of high-throughput sequencing and gene editing technologies in the identification and molecular breeding of the trait-related genes in orchids provides us with a representative trait-improving gene as well as some mechanisms worthy of further investigation. In addition, gene editing has promise for the improvement of orchid genetic transformation and the investigation of gene function. This knowledge may provide a scientific reference and theoretical basis for orchid genome studies.

## Introduction

The Orchidaceae family of monocotyledonous plants have the second-largest members after Compositae. This family contains over 750 genera and nearly 28,000 species (Zhang et al., 2017). Conventional orchids could be classified into five subfamilies (*Apostasioideae*, *Vanilloideae*, *Cypripedioideae*, *Epidendroideae*, and *Orchidoideae*) by their morphology and anatomy (Lu et al., 2019). The habitat of wild orchids has been gravely affected by natural and manmade factors. Many endangered species are on the edge of extinction due to indiscriminate gathering. The current protection efforts for orchids include the construction of nature reserves and genetic resource nurseries, as well as seed-preservation and *in vitro* tissue culture (Williams et al., 2018). Although this act ensures a huge number of original germs, the seedlings degenerate and eventually lose their ability to differentiate during the subculture processes, which makes it difficult to maintain the original genetic background. Besides, most orchids are cross-pollinated, and artificial pollination is considered essential in most cases (Suetsugu, 2015). Because of their huge species diversity and significant economic value, orchids have been the focus of study in botany and ecology for many years. China has a long history of cultivating orchids and has bred numerous varieties. So far, 187 genera and 1500 species of wild orchids have been recorded, including some subspecies and varieties (Chase et al., 2015). There are still several ornamental wild orchids to be created, preserved, and exploited in nature. In addition to its high economic and ornamental value, the orchid also has a profound historic origin. In Chinese traditional culture, the orchid referred to be one of the “four gentlemen among the flowers,” the others being the *Prunus mume*, *Chrysanthemum morifolium*, and *Sasa pygmaea* (Li et al., 2021).

Before the emergence of molecular-assisted breeding, distant hybridization was one of the most commonly used methodology for fertilizing orchids. In recent years, high-throughput sequencing technology and gene editing have been widely applied in the molecular biology, genomics, and discovery of trait-related genes in orchids, as well as modern genetic engineering breeding (Paun et al., 2010; Hsiao et al., 2021; Hsu et al., 2022; Li et al., 2022a). Whole genome sequencing of non-model organisms is now common due to the rapid advancement and lower cost of next-generation sequencing. The draft genome of *Phalaenopsis equestris*, a tropical epiphytic orchid that is normally utilized as a parent species in orchid breeding, was the first real achievement (Cai et al., 2015). Due to the fast development of ultralong sequencing and new assembly algorithms, whole-genome shotgun sequencing and single molecule sequencing have been done on even more orchid species, such as *Dendrobium officinale*, *Dendrobium catenatum*, *Dendrobium huoshanense*, *Phalaenopsis* ‘KHM190, *Phalaenopsis aphrodite*, *Gastrodia elata*, *Vanilla planifolia*, *Apostasia shenzhenica*, *etc.*  (Yan et al., 2015; Huang et al., 2016; Zhang et al., 2016; Zhang et al., 2016; Zhang et al., 2017; Chao et al., 2018; Yuan et al., 2018; Hu et al., 2019; Han et al., 2020; Niu et al., 2021). The growing number of orchid species with high-quality genomes and the use of advanced genetic analysis tools make it much easier to study the functional genes, especially those that are of interest for molecular breeding. The new advancement of genome editing technologies, such as the CRISPR/Cas9 system, is beneficial to this continuing endeavor (Wang et al., 2021). Depending on many defined gene transformation systems in orchids, the CRISPR/Cas9 tool has been effectively implemented in *P. equestris* by having to take tiny insertion/deletion or reversal mutations into target genes or perhaps the establishing kilobase-scale deletions of genes of interest. (Kui et al., 2017; Tong et al., 2020; Li et al., 2022b).

The market for orchids has expanded in size and diversity as a result of economic globalization, driving scientists and biologists to develop new varieties with distinctive looks, improved adaptability, and premium features (Li et al., 2021). Traditional breeding, despite being time- consuming, is always the predominant means of orchid cultivation. Because of the limitations and inefficiencies of the traditional approaches, hybridization and mutagenesis can not be used to get some desirable traits, like the spotted blooms and foliage of a single plant. Agrobacterium-mediated transformation and particle bombardment methods have been routinely used in transgenic molecular breeding, leading to significant progress in horticultural development (Liau et al., 2003; Men et al., 2003; Hsing et al., 2016; Khumkarjorn et al., 2017; Lin et al., 2018; Kayika Febryanti et al., 2020; Setiawati et al., 2020). Our understanding of orchid reproductive biology will undoubtedly change as a result of these efforts to enhance orchid genome-editing tools and the power of large-scale genome sequencing, which will enable us to better understand the inherent roles of orchid genes and changes to genes of interest for desired blooming and floral features (Molla and Yang 2019; Nopitasari et al., 2020; Tong et al., 2020; Guo et al., 2022). Here, we systematically summarized the studies on orchid genomes, including plastid genomes, especially the molecular evolution of orchids based on high-throughput sequencing technology and the identification and functional studies of trait-related genes. In addition, the application of gene editing and genetic transformation technologies in orchids was also discussed in detail.

## Genome size and ploidy analysis of the orchid

Ten years ago, only bacterial artificial chromosome (BAC) end sequences were used in genetic investigations of *Phalaenopsis* orchids. Short sequences can be used as molecular markers to assist in gene mapping and the construction of genetic maps. These sequences contained several repetitive DNA and SSR markers (Hsu et al., 2011). Cytogenetic evidence is only available for few orchid species (Felix and Guerra, 2010). *Cattleya*, *Cymbidium*, *Dendrobium*, *Oncidium*, *Phalaenopsis*, *Paphiopedilum*, *Vanilla*, and *Vanda* are examples of commercially significant genera that are valuable in floriculture, medicinal, and food condiments (da Rocha Perini et al., 2016; Vilcherrez-Atoche et al., 2022). Chromosomal counting and nuclear DNA content estimation with flow cytometry (FCM) are the most popular techniques employed for polyploid identification in these orchids (Younis et al., 2013; Mohammadi et al., 2021). Using flow cytometry, the genetic traits and types of endoreplication of 149 orchid species were compared. The variations in genome size and particularly in GC contents were inextricably bound with evolutionary transitions from the conventional mode of endoreplication to partial endoreplication (Trávníček et al., 2019). In eukaryotic species, nuclear genome size is an inherited quantitative feature with both biological and practical relevance. Genome size, karyotype, and nucleobase composition vary significantly across angiosperms, with potential adaptive consequences. A systematic analysis of the major plant families could help us understand the biological significance of the huge differences in genome size within plants. Several studies have assessed C-values in 48 orchid species in order to analyze the distributions of nuclear DNA quantities and identify tissues suited for accurate estimations of nuclear DNA content (Trávníček et al., 2015; Rewers et al., 2021). Additional analysis on the size of the genomes of *Pleurothallidinae* species showed that those with partial endoreplication (PE) had much bigger genomes and that the number of genomic repeats was closely linked to the size of the non-endoreplicated part of the genome (Chumová et al., 2021). According to previous investigations on the variation of Apostasioideae genome size, the predicted 1C-values vary from 0.38 pg in *Apostasia nuda* to 5.96 pg in *Neuwiedia zollingeri* var. javanica, a roughly 16-fold difference. The genome sizes of the two genera did not overlap. *Apostasia* had much smaller genomes than *Neuwiedia*, which suggested that smaller genomes were common in the Apostasioideae subfamily (Jersáková et al., 2013). The genome of *Apostasia ramifera* showed the population size histories of many orchid species, as well as a continual fall in population size in seven orchid genomes (Zhang W. et al., 2021). Some research had shown that the incorporation of LTR retrotransposons Orchid-rt1 and Gypsy1 into *Phalaenopsis* genomes might be linked to genome size growth (Hsu et al., 2020). Genome size is also linked to cellular and developmental characteristics. The evolutionary connection between genome size, floral lifespan, and labellum epidermal cell size in *Paphiopedilum* revealed that genome size was connected to floral duration but negatively relevant to labellum epidermal cell size (Zhang and Zhang, 2021).

In addition to flow cytometry, k-mer analysis-based genome survey sequencing is also a common method for estimating genome size. It has the advantages of high-throughput sequencing, high speed, and large amounts of data, which can quickly determine the size and heterozygosity of the genome (Lee et al., 2017). The k-mer depth values are often derived from the curves used to estimate genome size. Through the distribution of the k-mer curve, the genomic characteristics are estimated, and the ratio of the heterozygous peak to the homozygous peak is calculated to obtain the heterozygous rate (Jersáková et al., 2013). For determining the size of orchid genomes, k-mer analysis based on the Illumina Hiseq sequencing platform has been widely applied. The genome of *C. ensifolium* was evaluated using 17-mer analysis, which indicated the genome size and heterozygozity to be 3.56 Gb and 1.40%, respectively (Ai et al., 2021). The estimated genome size of *G. menghaiensis* based on k-mers is 0.98 Gb, with 0.1% heterozygosity and high repeats. The 17-mer distribution is Poisson-distributed and is dependent on the properties of the genome (Jiang Y. et al., 2022). Using k-mer distribution analysis, the genome size, heterozygozity, and repetitive ratio of *D. officinale* were determined. The largest peak of 17 k-mer frequency was seen at a depth of 90, allowing the determination of the genome size, heterozygosity, and repetitive ratio (Niu et al., 2021).

The development of the orchid industry benefits greatly from the ploidy identification of orchid germplasm resources. Chromosomal and cytological investigations revealed that *Cymbidium* species contained a prevalence of 40 chromosomes along with variations found in *C. serratum* (41, 43, 60, and 80). From the earliest polyploids recorded at the beginning of the 20th century, it has been feasible to create a number of *Cymbidium* polyploid cultivars through biological and artificial approaches (Xie et al., 2017). Since then, *Cymbidium* cultivars have been known to be diploids, triploids, and tetraploids with distinct chromosomal morphology (Younis et al., 2013). About 75.8% of *C. hybridum* cultivars harbor polyploids, indicating a link between the intentional or unintentional selection of polyploids instead of diploids for superior features (Vilcherrez-Atoche et al., 2022). The majority of *Dendrobium* species contained 38 chromosomes, with the exception of *D. leonis* and *D. dixanthum*, which both have 40 chromosomes (Zheng et al., 2018). The majority of *Phalaenopsis* species have 38 chromosomes, with the exception of the Aphyllae, which has only 34 or 36 chromosomes (Lee et al., 2017). However, a significant heterogeneity of genome size was detected among species and hybrids within this genus (Chang et al., 2006; Lee et al., 2017). *Phalaenopsis* cultivars have a wide range of chromosomal numbers (38, 57, and 76 more), indicating polyploidy. Flower gardening traditionally employs *Phalaenopsis* hybrid cultivars. Only one diploid cultivar has been documented, whereas over 80% of tetraploid cultivars have 76 chromosomes (Lee S. Y. et al., 2020). The domination of commercial tetraploid cultivars demonstrates the relevance of polyploidy in the development of better *Phalaenopsis* cultivars. These tetraploid species are implemented as parentals to create subgroups of *Phalaenopsis* cultivars with the goal of achieving desirable colors for commercial purposes (Bolaños-Villegas and Chen, 2007; Li et al., 2012). *Vanda*, like *Dendrobium* and *Phalaenopsis*, has 38 chromosomes and naturally occurs in tetraploid and hexaploid species (Khan et al., 2019; Liu et al., 2020). In *Oncidium*, it is assumed that x = 7 is the basic number of chromosomes, but unlike other genera, there is a huge chromosomal variation across species, with the majority exhibiting polyploidy (Su et al., 2013).

## Evaluation of gene duplication events under high-quality genome sequencing in orchid

**Figure 1:**
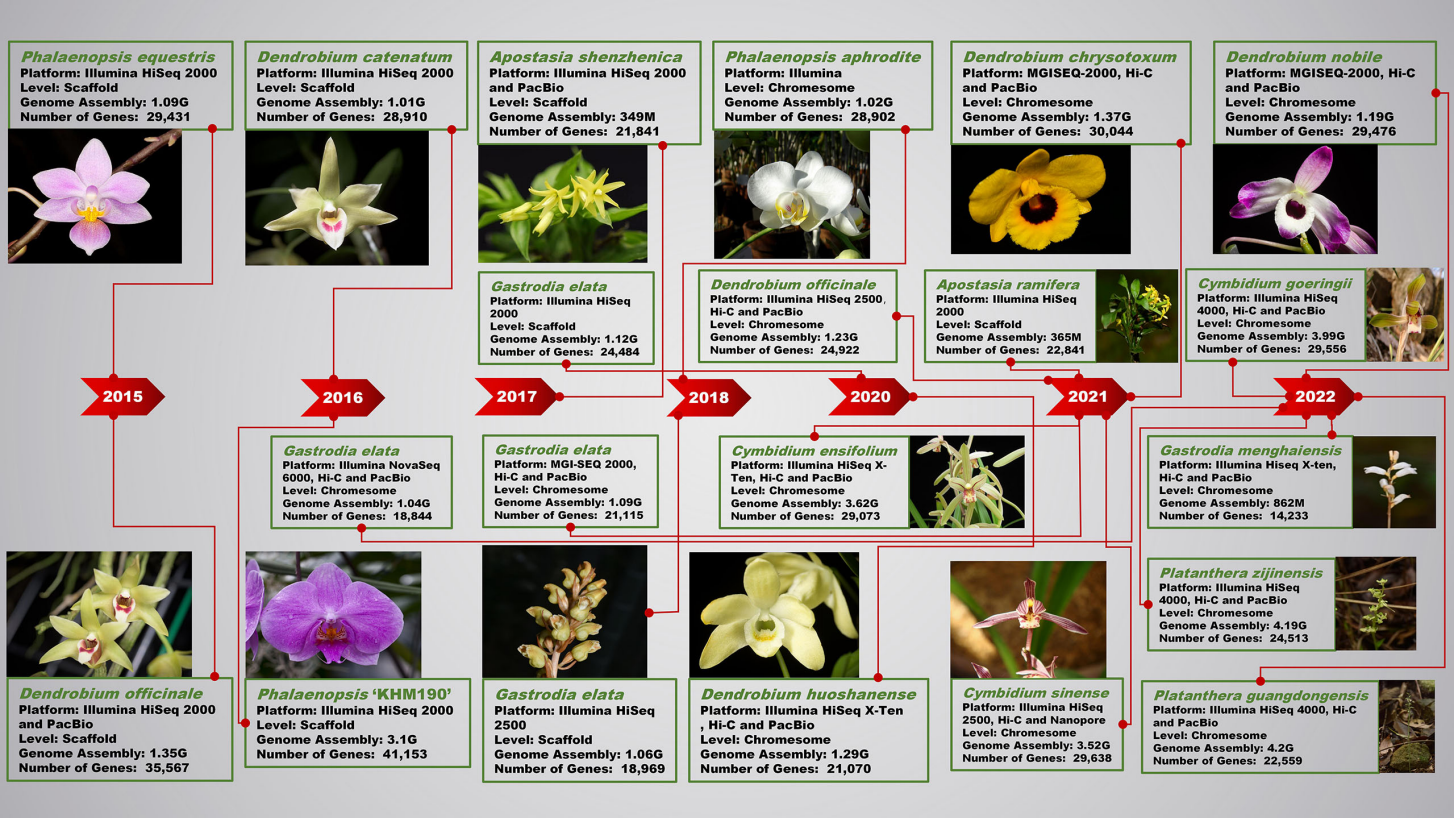
Research progress of next-generation sequencing and third-generation sequencing technology in orchid genomes.

The continuity and integrity of model plant genomes have also been greatly improved due to the continuous development of genome research and the improvement of sequencing technology. The orchid genome has gone through the draft genome obtained by ordinary next-generation sequencing, to the chromosome-level genome assembled by PacBio or ONT sequencing technology combined with Hi-C, and then to the near complete genome obtained by ONT (N50>50Kb) assembly (**Figure 1**). By combining ONT ultra-long and PacBio HiFi techniques, those gap-free genomes assembled at telomere to telomere (T2T) level will be a new direction in the future. The whole genome sequencing of the *A. shenzhenica* helps us better understand the origins and evolution within subfamilies (Zhang et al., 2017). The whole genome duplication (WGD) that has occurred more than once in plant genomes is a noteworthy feature (Clark and Donoghue, 2018). Angiosperm genome sequences provide information regarding polyploidy and genome evolution. By evaluating the prevalence of synonymous substitutions per synonymous site (Ks) throughout all paralogous genes and duplicated genes situated in synteny blocks based on the *Phalaenopsis* and *Dendrobium* genome sequences, two WGDs were projected to have evolved in the *D. catenatum* lineage. (Zhang et al., 2016). The nearest WGD event is shared by *Dendrobium*, *Phalaenopsis*, and *Apostasia*, and it could have occurred near the Cretaceous-Paleogene (K/Pg) boundary. Peaks in older Ks distributions are thought to be an additional ancient WGD event shared by monocot ancestors (Cai et al., 2015; Zhang et al., 2016; Zhang et al., 2017). The draft genome sequencing revealed compelling evidence of a whole-genome duplication that all orchids share and that came right before their divergence (Zhang et al., 2017). The MADS-box family members may govern a wide spectrum of developmental events during orchid evolution. A chromosomal-scale genome and chromosome linkage groups of *P. aphrodite* were first created, which contributed to the variation in labellum and pollinium morphology and structures (Chao et al., 2018). A chromosome-scale genome assembly of *C. goeringii* suggested several new gene families, resistance-related homologs and variations within the *MADS-box* genes may regulate a wide set of developmental processes during adaptive evolution (Chung et al., 2022). A haplotype-resolved genome of *Bletilla striata* reveals its evolutionary relationship with other orchids, which have experienced an ancient WGD event shared with monocots and a recent WGD event within all orchids. The biochemical machinery of *B. striata* polysaccharide (BSP) biosynthesis indicated that MYB2 interacted physically with some BSP-regulated genes (Jiang L. et al., 2022). Partial endoreplication has been discovered across all *Vanilla* species. A chromosome-scaled genome of *Vanilla planifolia* showed that the genome size discrepancy was driven by the presence of PE (Piet et al., 2022).

Mycoheterotrophic and parasitic plants get some or all of the nutrients they need from other organisms. *Gastrodia* fungi are typically perennial, achlorophyllous orchids with a unique evolutionary mechanism for adaptability to a non-photosynthetic lifestyle. The genome of *G. elata* reveals the genetic basics of most adaptive changes in photosynthesis, leaf development, and plastid division (Chen S. et al., 2020). Comparative genomics studies revealed that *G. elata* and other completely heterotrophic species dropped nearly 10% of the conserved orthogroups, including those important for autotrophs (Xu et al., 2021). Photosynthesis, circadian clock, flowering control, immunity, food intake, and root and leaf growth are all governed by these orthogroups. Recent assembly of the *G. elata* genome also showed a strong contraction of genes which involved in multiple biosynthetic processes and cellular components but also an expansion of genes for some metabolic processes and mycorrhizal interactions (Bae et al., 2022). Many genes involved in arbuscular mycorrhizae colonization and biological interaction between *Gatrodia* and symbiotic microbes were identified in the genome of *G. menghaiensis* (Jiang Y. et al., 2022). The loss and conservation of symbiotic genes associated with the evolution of unique symbionts in plants were determined by analyzing a broad array of plant genome and transcriptomics data. A shared symbiosis network progressed at the same time as intracellular endosymbioses, from the primitive arbuscular mycorrhiza to the more recent ericoid and orchid mycorrhizae in angiosperms and ericoid-like connections in bryophytes (Radhakrishnan et al., 2020). The comparison of *Platanthera zijinensis* and *Platanthera guangdongensis* genomes indicated that mycoheterotrophy is linked to higher rates of gene loss and alternation, and that the deletion of most photoreceptor and auxin transporter genes might explain how fully mycoheterotrophic orchids look so different from other orchids. Some trehalase genes have grown, which makes sense since orchid non-endosperm seeds need carbohydrates from fungi to sprout when they are in the protocorm stage (Li M-H. et al., 2022; Minasiewicz et al., 2022).

*Dendrobium* is the second biggest genus in Orchidaceae. The first genome of a lithophytic orchid, *D. catenatum* (now recognized as *D. officinale*), showed wide duplication of genes associated with glucomannan synthase (Yan et al., 2015; Zhang et al., 2016). Recent assembly of the *D. officinale* genome has brought new insights into the evolution of this *Dendrobium* spp. (Niu et al., 2021). Our previous study released a chromosome-level assembly of the *D. huoshanense* genome with PacBio sequencing and Hi-C method (Han et al., 2020). A chromosome-scale reference genome of *D. chrysotoxum* was also obtained based on PacBio sequencing and Hi-C methods. The phylogeny of the *SWEET* gene family implied that gene expansion occurred in clade II may associated with fleshy stems rich in polysaccharides (Zhang Y. et al., 2021). *Cymbidium* is famous for its distinctive leaves, flower morphology, and pleasant aroma (Yang L. et al., 2021). The genome of *C. ensifolium* has undergone two WGD events, and the abnormal expression of *MADS-box* genes might be related to flower development and shape mutations (Ai et al., 2021). A chromosome- scale genome of *D. nobile* showed two polyploidization events occurred. The expression profile of *TPS* and *CYP450* genes suggested that the distinct distribution of *TPS-b* subclade may contribute to the species-specific alkaloid biosynthesis pathways (Xu et al., 2022). Finally, a phylogenetic tree was constructed based on single-copy genes to better demonstrate the evolutionary relationship between orchid species (**Figure S1**).

The associated mapping method performed statistical analyses to discover the importance of the relationship between genetic variants and polymorphism in a group of individuals with genetic variations (Ogura and Busch, 2015). Large-scale resequencing has been broadly used for gene mapping of crop quality traits and differential analysis of SNP loci within genes. However, investigations for genome-wide association studies (GWAS) based on genotyping-by-sequencing (GBS) have received less attention in orchids. Through NGS technology, a large number of SNP markers have been found through sequencing to create a high-density genetic map. A total of 691,532 SNP sites were identified to generate a genetic linkage map for marker-assisted selection breeding by resequencing *Phalaenopsis pulcherrima* and denovo sequencing of *Phalaenopsis* ‘KHM190’ (Huang et al., 2016). Species-specific markers could help to identify unknown intraspecies and validate the parentage of interspecifc hybrid offspring. Genomics-based diversity analysis of *Vanilla* species indicated that the value of the GBS approach to interpret diversity in *Vanilla* collections has been demonstrated to be the paternal parent of hybrids more efficiently than other methods (Hu et al., 2019). The interspecific hybridization of *D. nobile* and *Dendrobium wardianum* was used to construct a population with 100 F1 individuals (Li J. et al., 2019). A total of 331,642 SNP markers were obtained, 9645 of which were used to build a high-density genetic map with 19 linkage groups, and three QTLs identified may be associated with stem length and diameter. The genetic diversity and variations among *Dendrobium* mutants and common *Dendrobium* cultivars were compared based on SNPs by GBS (Ryu et al., 2019). A total of 517,660 SNPs were identified, 37,721 of which were used to discriminate the differences across *Dendrobium* genotypes. 129 accessions were collected from 10 wild cultivated populations to explore the genetic diversity and population structure of *D. nobile* in China (He et al., 2022). Approximately 830,000 SNPs were obtained and used for genetic variation analysis. The recent completion of the chromosome-level assembly of the *D. officinale* genome provides a reliable data basis for its genetic background and breeding improvement. Niu and his colleagues performed *D. officinale* resequencing to conduct a GWAS investigation on 38 cultivars and five related species (Niu et al., 2021). A total of 13 GWAS loci were identified to associate with some morphologic traits.

## Sequencing and evolution of the chloroplast genome in orchid

The chloroplast genome (cp) contains more conserved structures than the nuclear and mitochondrial genomes, which is beneficial for systematics and species identification. Studies on the chloroplast genomes of Orchidaceae have remained prominent in recent years (**Table 1**). The chloroplast genomes of *D. officinale* and *Cypripedium macranthos* were compared, and there were parallels in structure as well as gene order and content, but there were differences in the organization of the inverted repeat/small single-copy junction and *ndh* genes (Luo et al., 2014). Since *ndh* genes are truncated or excluded in the cp genomes of some autotrophic Epidendroideae orchids, some studies had mentioned that these gene deletion events are independent (Lin et al., 2015). By comparing 53 cp genomes, it was indicated that the expansion of inverted repeats in *Paphiopedilum* and *Vanilla* is also associated with a loss of *ndh* genes (Niu et al., 2017b). *Bulbophyllum Thou.* is one of the biggest genera with over 2,000 species, found in rainforest regions (Gamisch and Comes, 2019). Long-term geographic isolation exposed Asian and South American *Bulbophyllum* cp genomes to varying selective pressures (Yang et al., 2022). Besides the *Bulbophyllum* orchids, plastid genome sequencing has been reported for a large number of *Dendrobium* species, which are commonly used for phylogenetic studies and variety authentication (Zhang et al., 2018; Wu X.-Y. et al., 2019; Liu et al., 2021). *Phalaenopsis* orchids are another orchid species that has received significant interest (Chang et al., 2006; Kim et al., 2016; Wang et al., 2019; Xia et al., 2021). *Paphiopedilum*, also known as slipper orchid, is well-known for its large, specialized lip, as well as its lovely flowers and colors. The cp genome of many *Paphiopedilum* orchids was investigated to provide the phylogenomic analysis of this species and its relatives (Zhao et al., 2019; Tang F. L. et al., 2020; Hu et al., 2022). Furthermore, the cp genomes of some other orchid genera or subtribes have been published, including *Pelatantheria scolopendrifolia*, *Cymbidium ensifolium*, *Eulophia flava*, *Calanthe arcuat*a, and *Coelogyne fimbr* (Yun et al., 2018; Bertrand et al., 2019; Jiang et al., 2019; Li H. et al., 2019; Zhong et al., 2019). These results are important for figuring out how chloroplasts have changed over time and how gene structures vary in orchids (Zeng et al., 2007). A phylogenetic tree of 58 representative orchid species was constructed to investigate the relationship of cp genomes within subfamilies or subtribes (**Figure S2**). The results also revealed that these varieties could be classified into five subfamilies, with the majority of individuals belonging to the Epidendroideae and Orchidoideae.

**Table 1:** Features of representative plastid genomes in orchidaceae.

Subfamily	Taxon	Total length (bp)	Large single copy (LSC)	Inverted repeat (IR)	Small single copy (SSC)	Protein-coding genes	Accession	Reference
Epidendroideae	*Dendrobium officinale*	152,221	85,109	26,298	14,516	76	KC771275	Luo et al., 2014
	*Pelatantheria scolopendrifolia*	146,971	86,096	24,570	11,735	72	MG752972	Yun et al., 2018
	*Dendrobium bellatulum*	152,107	85,061	26,297	14,503	83	MG595965	Zhang et al., 2018
	*Dendrobium comatum*	158,008	85,592	27,032	18,352	87	MZ666386	Liu et al., 2021
	*Dendrobium nobile*	152,018	84,944	26,285	14,504	79	KX377961	Konhar et al., 2019
	*Cymbidium ensifolium*	150,257	85,110	25,692	13,761	78	MK841484	Jiang et al., 2019
	*Cymbidium mastersii*	155,362	84,465	25,125	20,647	80	MK848042	Zheng et al., 2019
	*Cymbidium floribundum*	153,998	84,725	25,132	19,009	80	MK848043	Zhang G. Q. et al., 2019
	*Cymbidium hookerianum*	155,447	84,186	26,711	17,839	78	MT800927	Wei et al., 2021
	*Cymbidium aloifolium*	157,328	85,793	26,829	17,877	78	MN641752	Chen J. et al., 2020
	*Cymbidium floribundum* var. *pumilum*	155,291	84,415	26,696	17,484	80	MN173778	Ai et al., 2019a
	*Cymbidium sinense* x *C. goeringii*	150,149	84,987	25,691	13,780	75	MN532117	Choi et al., 2020
	*Cymbidium dayanum*	155,408	84,189	26,614	17,991	76	MW160431	Du et al., 2021
	*Cymbidium bicolor*	156,528	85,907	26,703	17,215	78	MN654912	Hu et al., 2020
	*Dendrobium longicornu*	160,024	88,075	25,403	21,143	80	MN227146	Wu X.-Y. et al., 2019
	*Calanthe arcuata*	158,735	87,348	26,449	18,489	88	MK934523	Zhong et al., 2019
	*Danxiaorchis singchiana*	87,931	42,575	13,762	17,831	36	MN584923	Lee S. Y. et al., 2020
	*Coelogyne fimbriata*	158,935	87,444	26,374	18,743	91	MT548043	Yue et al., 2020
	*Pleione maculata*	158,394	86,603	26,646	18,499	89	MW699846	He et al., 2021
	*Pleione bulbocodioides*	159,269	87,125	26,716	18,712	81	KY849819	Shi et al., 2018
	*Pleione chunii*	158,880	87,259	26,465	18,691	87	MK792342	Wu S. et al., 2019
	*Hexalectris warnockii*	119,057	66,903	17,332	17,490	38	MH444822	Barrett and Kennedy, 2018
	*Arundina graminifolia*	159,482	87,285	26,813	18,581	88	MN171408	Ai et al., 2019b
	*Eulophia zollingeri*	145,201	81,566	25,272	13,091	86	MG181954	Huo et al., 2018
	*Dendrobium thyrsiflorum*	160,123	88,001	25,490	21,142	80	MN306203	Pan et al., 2019
	*Liparis vivipara*	158,329	85,950	27,043	18,293	77	MK862100	Zhang D. et al., 2019
	*Liparis bootanensis*	158,325	86,584	26,700	18,341	83	MN627759	Liu, 2020
	*Tainia dunnii*	158,305	86,819	25,244	20,998	88	MN641754	Xie et al., 2020
	*Gomesa flexuosa*	147,764	83,579	25,757	12,671	73	OL692830	Mo et al., 2022
	*Geodorum densiflorum*	149,468	85,070	25,554	13,290	76	MT153204	Tang J. M. et al., 2020
Orchidoideae	*Phalaenopsis aphrodite*	148,964	85,957	25,732	11,543	65	AY916449	Chang et al., 2006
	*Phalaenopsis* ‘Tiny Star’	148,918	85,885	25,755	11,523	70	KJ944326	Kim et al., 2016
	*Phalaenopsis equestris*	148,959	85,967	25,846	11,300	75	JF719062	Jheng et al., 2012
	*Phalaenopsis wilsonii*	145,096	84,688	24,787	10,834	73	MW194929	Fan et al., 2021
	*Ophrys aveyronensis*	146,816	80,495	16,309	16,309	79	MN120441	Bertrand et al., 2019
	*Phalaenopsis lowii*	146,834	84,469	25,944	10,477	76	MN385684	Wang J. Y. et al., 2019
	*Vanda subconcolor*	149,490	85,691	25,912	11,975	74	MT180955	Liu et al., 2020
	*Phalaenopsis wilsoniii*	145,373	84,996	24,855	10,668	76	MW218959	Xia et al., 2021
	*Habenaria ciliolaris*	154,544	84,032	25,455	19,602	133	MN495954	Chen et al., 2019
	*Satyrium nepalense* var. *ciliatum*	154,418	83,475	26,715	17,513	79	MN497244	Ma et al., 2019
	*Spiranthes sinensis*	152,786	83,446	25,701	17,938	78	MK936427	Fan and Huang, 2019
	*Anoectochilus roxburghii*	152,802	82,641	26,364	17,433	81	KP776980	Yu et al., 2016
	*Nothodoritis zhejiangensis*	143,522	83,830	24,464	10,764	74	MW646088	Yang L. et al., 2021
	*Goodyera foliosa*	154,008	83,248	25,045	20,670	80	MN443774	Zhou et al., 2019
Cypripedioideae	*Cypripedium macranthos*	157,050	85,292	26,777	18,285	79	KF925434	Luo et al., 2014
	*Paphiopedilum hirsutissimum*	154,569	85,198	34,344	683	79	MN153815	Zhao et al., 2019
	*Paphiopedilum emersonii*	162,590	87,852	36,934	870	81	MT648789	Tang F. L. et al., 2020
	*Paphiopedilum gratrixianum*	157,292	87,252	34,106	1,828	68	MW284890	Hu et al., 2022
	*Paphiopedilum barbigerum*	156,329	86,056	34,214	1,845	80	MN153814	Li M. et al., 2019
	*Paphiopedilum parishii*	154,689	86,863	32,690	2,446	82	MW528213	Kao et al., 2021
	*Paphiopedilum bellatulum*	156,567	88,243	32,336	3,652	76	MN315107	Peng et al., 2020
	*Paphiopedilum spicerianum*	157,292	87,252	34,106	1,828	71	MT683624	Ge et al., 2020
Apostasioideae	*Apostasia wallichii*	156,126	83,035	26,452	20,187	79	LC199394	Niu et al., 2017a
	*Apostasia ramifera*	157,518	86,353	27,360	16,445	87	MT864006	Zheng et al., 2021
	*Apostasia shenzhenica*	153,164	86,167	27,510	11,977	75	MK370661	Li Y. et al., 2019
	*Neuwiedia singapureana*	161,068	89,031	26,991	18,058	79	LC199503	Niu et al., 2017a
Vanilloideae	*Cyrtosia septentrionalis*	96,859	58,085	10,414	17,946	38	MH615835	Kim et al., 2019
	*Vanilla shenzhenica*	151,537	87,487	22,439	19,172	69	MK962478	Li T. Z. et al., 2019
	*Vanilla pompona*	148,009	86,358	29,807	2,037	75	MF197310	Amiryousefi et al., 2017

Orchids have undergone more independent transitions to heterotrophy than any other land plants. Another interesting fact is that some heterotrophic orchids lose photosynthesis and autotrophy-related genes on chloroplasts throughout evolution, which provides an excellent opportunity to explore the effects of shifting selective regimes on genome evolution (Li M.-H. et al., 2022). As a consequence of the relaxation of functional restrictions on photosynthesis, certain heterotrophic plants, such as mycoheterotrophs and parasites, exhibit enormous gene losses. The comparative genomics of 12 tribe *Neottieae* orchids indicated that genes related to the NAD(P)H dehydrogenase complex, photosystems, and RNA polymerase were functionally lost many times (Feng et al., 2016). A phylogenetic analysis of 26 full plastome sequences from *Epidendreae* suggested that photosynthesis-related genes such as the atp complex had undergone severe gene loss (Lee S. Y. et al., 2020). Numerous investigation have identified evidence of fast plastome degradation in heterotrophic orchids based on the accumulation of pseudogenes and substantial deletions (Barrett and Kennedy, 2018; Barrett et al., 2019; Kim et al., 2019). Infraspecific analysis of the plastome evolution of leafless *Corallorhiza* revealed that considerable changes in plastome size and functional gene composition occurred in just a few million years as a consequence of decreasing selection constraints on photosynthesis (Barrett et al., 2018).

## Functional genomics study of orchid development and breeding

Orchid genome sequencing initiatives and other cuttingedge technologies, such as genome editing tools are undoubtedly facilitating molecular genetic studies on orchid reproductive development. The genome sequencing of the tropical epiphytic orchid *P. equestris*, which provide an important resource for beginning to explore orchid diversity and evolution at the genome level, was a significant step forward in orchid genome study (Cai et al., 2015). It is now possible to identify and compare gene families that might have new functions across the whole genome with the availability of whole genome sequences (Lin et al., 2016; Cao et al., 2019; Chen T. C. et al., 2020; Song et al., 2021). As most orchid plants contain both C4 metabolism and CAM, phosphoenolpyruvate carboxylase (PEPC) plays an important role in photosynthetic performance and CO_2_ efficiency. For green plants, especially CAM plants, little is known about the evolutionary history of the *PEPC* gene family. Using high-throughput sequencing and comprehensive phylogenetic analysis, the results indicated that CAM or C4-related PEPC may originate from the PPC-1M1 clade. The WGD event was responsible for the increment of *PEPC* gene copies (Deng et al., 2016). The plant-specific YABBY TFs regulate leaf polarity. Two *DROOPING LEAF/CRABS CLAW* (*DL/CRC*) genes were discovered in *P. equestris*, where *PeDLs* have demonstrated conserved function in floral meristem and carpel development (Chen et al., 2021). Protocorm-like bodies (PLBs) are commonly utilized in orchid micropropagation (Ren et al., 2020). According to certain research, SHOOT MERISTEMLESS (STM)-dependent organogenesis is required for PLB formation (Fang et al., 2022). Overexpression of *PaSTM* improved the regeneration from vegetative tissue-based explants of *Phalaenopsis*.

Moreover, many studies have demonstrated that *MADS-box* family genes control flower formation and morphogenesis (Teo et al., 2019). So far, a total of 51, 56, and 63 putative ones have been noticed in *P. equestris*, *P. aphrodite* and *D. catenatum*, respectively (Cai et al., 2015; Zhang et al., 2016; Chao et al., 2018). Despite having fewer *MADS-box* genes than *Arabidopsis* (107 genes) and rice (80 genes), orchids have more *MADS-box* genes involved in floral organ production (Leseberg et al., 2006). This distinction suggests that higher *MADS-box* gene diversity might be connected with highly specific floral morphological traits in orchids (Cai et al., 2015; Chao et al., 2018). This hypothesis is backed further by the fact that the number of *MADS-box* genes differs across Apostasioideae and the other orchid subfamilies. *A. shenzhenica*, a member of the Apostasioideae subfamily, yields solanum-type flowers with undifferentiated lips and somewhat simple gynostemia (Chen et al., 2012). *A. shenzhenica* contains fewer B-class AP3-clade and E-class *MADS-box* genes than *Dendrobium* and *Phalaenopsis* (Cai et al., 2015; Zhang et al., 2016). Notably, all modern orchids have shared a WGD event, which may be related to their diversification (Zhang et al., 2017; Yuan et al., 2018). The B-class AP3-clade and E-class genes may have increased just after WGD in the common ancestor of all orchids. Nevertheless, their paralogous genes may have been eliminated in *Apostasia*, culminating in a reversion to an earlier form with the plesiomorphic bloom (Zhang et al., 2017).

In the long term, the orchid breeding paradigm has seen the transition from conventional selection to cross-breeding, from molecular-assisted breeding to gene editing breeding (Li et al., 2021). Except for some self-incompatible species, the hybrid progeny preserve the parents’ superior genetic features (Niu et al., 2018). However, the fertility of the hybrid combination and the genetic instability of the embryo after fertilization, the mapping of important agronomic traits and the selection of homozygotes are challenges (Su et al., 2019). Among them, seed germination is closely related to hybridization efficiency. When hybrid seeds are obtained, a proper cultivation technique is required to maintain the population. *In vitro* cultivation is a common method of seed propagation that has been used in the cultivation of numerous orchid species (Gao et al., 2020). The major goals of *in vitro* propagation are hybrid gex and a reduced breeding cycle. Mutagenesis breeding is also broadly applied for selecting elite crop and horticultural plant varieties. Many orchid varieties, including *Dendrobium*, *Phalaenopsis*, *Cymbidium*, *Oncidium*, *etc.*, have successfully undergone polyploid breeding by colchicine induction (Vilcherrez-Atoche et al., 2022). The high heterozygosity of orchids can lead to an increase in the perceived mutation rate and result in a flurry of good mutation types. However, unpredictable mutations can occur throughout the genome, and those negative mutations may occur, with only minor changes frequently achieved (Su et al., 2019). Molecular marker-assisted breeding is fast, efficient, and independent of environmental factors. Techniques such as AFLP, RFLP, SSR, RAPD, *etc*. are regularly employed to identify trait-related differential sequences (Poczai et al., 2013). These markers, when combined with function annotation given by unigenes, enable the identification of candidates with specific roles. Moreover, the completion of large-scale chromosome-level genomes lays the foundation for gene editing breeding and precise breeding based on features.

## Discussion

Polyploidy is the driving force behind species adaptation, diversity, and genome evolution. Some superior orchid cultivars are produced through chromosomal polyploidy in the domain of horticulture (Vilcherrez-Atoche et al., 2022). Domestication and polyploidy have a close link since polyploid plants are randomly selected for their greater vigor, and consequently, polyploid species are more profitable and attractive for domestication than wild ones. The size of a genome is mostly determined by endoreplication and LTR retrotransposon insertion during expansion (Chumová et al., 2021). Initially, FCM and k-mer analysis was used to calculate the size of these genomes. Large-scale tandem duplication and segmental duplication within the chromosome drive the generation of new genes and species evolution (Clark and Donoghue, 2018). In most cases, orchids underwent WGD more than once, including a historical WGD event and a recent WGD event shared by all orchids. There are both mycoheterotrophic and parasitic orchids, in addition to the vast majority of ornamental orchids. The loss and survival of symbiotic genes related to the evolution of specific symbionts span from the ancestral arbuscular mycorrhiza to the recent ericoid and orchid mycorrhizae (Barrett et al., 2019; Gao et al., 2020). Fully mycoheterotrophic orchids look very different from other orchids. This might be due to the loss of most of their photoreceptor and auxin transporter genes. Large-scale resequencing has been utilized to pinpoint key genes or chromosomal regions linked with some trait characteristics. GWAS based on GBS has sparked a lot of interest in several orchids. Some valuable SNP markers are widely applied to discriminate against orchid varieties (Kumagai et al., 2019). Furthermore, a small single-copy region in the cp genome of *Paphiopedilum* lost a large number of sequences, implying its significance in adaptive evolution (Trávníček et al., 2015). In this study, a phylogenetic tree of 58 orchid species was constructed to investigate the relationship of cp genomes within five subfamilies. The major sequenced species are those designated as Epidendroideae and Orchidoideae. MADS-box transcriptional factors are one of the most studied gene families in orchids, with evidence that they are involved in the regulation of various developmental processes as well as responses to environmental stimuli (Teo et al., 2019). The biological functions of these MADS-box proteins and the mechanisms by which they contribute to flowering or floral organ development are detailed. The molecular mechanisms underpinning flowering and floral development can be exploited for both traditional orchid breeding and targeted manipulation for desired blooming features.

Despite recent advancements in the field of orchid reproductive development, molecular genetic studies of flowering initiation and development continue to lag behind those in other model plants due to a number of bottlenecks. These included the prolonged vegetative stage, the inefficiency of established genetic transformation systems, and available data on genome sequences (Wang et al., 2017). Consequently, the majority of studies on orchid reproductive development have concentrated on genes that are homologs of other well-known genes in model plants.The duplication of genes in the genomes of some orchids may be beneficial for the inheritance of specific characteristics that contribute to the adaption to various environments. Furthermore, clarifying the inherent roles of the key genes in homologous orchid transgenic systems is critical (Hsing et al., 2016; Zhang et al., 2017). This technique involves the ongoing development of a few orchid-specific technical platforms, such as *in vitro* tissue culture, gene transformation, and genome editing tools (Hsiao et al., 2011; Li et al., 2022b). Many recent studies on the crop pan-genome have successfully identified core genes, individual-specific genes, and structural variation between many subspecies, providing new insights into the genetic underpinning of intricate biological characteristics (Liao et al., 2004; Li et al., 2021). A pan-genome encompasses more genetic variation within plants than a single reference genome. Therefore, another research hotspot of orchids may be concentrated on pan-genome and next-generation breeding technologies under the genetic background of different species (Tsai et al., 2017). Together, these efforts and the ever-improving use of multi-omics techniques to find specific molecular markers linked with morphological changes in orchid reproductive development will pave the way to figure out the molecular basis of specialized orchid reproductive processes.

## Author contributions

CS, FZ, and YC discussed the writing plan. CS, YW and DM wrote the draft manuscript. CS, MM, and PPW edited the manuscript. FZ and CS acquired the funding. All the authors have read and approved the submitted version.

## Funding

This work was supported by Anhui Province Postdoctoral Fund (2020B454), High-level Talents Research Initiation Fund of West Anhui University (WGKQ2022025), Postdoctoral Fund of West Anhui University (WXBSH2019001), and Anhui Provincial Administration of Traditional Chinese Medicine Project (2020zcyb09).

## Acknowledgments

We apologize to those authors whose excellent work could not be cited because of space restrictions.

## Conflict of interest

The authors declare that the research was conducted in the absence of any commercial or financial relationships that could be construed as a potential conflict of interest.

## Publisher’s note

All claims expressed in this article are solely those of the authors and do not necessarily represent those of their affiliated organizations, or those of the publisher, the editors and the reviewers. Any product that may be evaluated in this article, or claim that may be made by its manufacturer, is not guaranteed or endorsed by the publisher.

## Supplementary material

Supplementary Figure 1 The phylogenetic tree of 11 orchid species with publicly available protein sequences based on the identified single-copy genes. *A. thaliana* was regarded as an outgroup. The tree was visulized by the iTOL online service (https://itol.embl.de/).

Supplementary Figure 2 The maximum-likelihood (ML) tree of 58 Orchidaceae species based on the chloroplast genomes. Alignments of the cp genomes were performed using MAFFT (*v*7.505) based on the FFT-NS-2 method (https://mafft.cbrc.jp/alignment/software/). The Archaeopteryx.js tool was used to display the ML tree (https://sites.google.com/site/cmzmasek/home/software/archaeopteryx).
