# Supplementary figures and images for: In-depth analysis of genomes and functional genomics of orchid using cutting-edge high-throughput sequencing
Source: Front Plant Sci. 2022 Sep 23;13:1018029. doi: 10.3389/fpls.2022.1018029 (PMC9539832; doi:10.3389/fpls.2022.1018029)

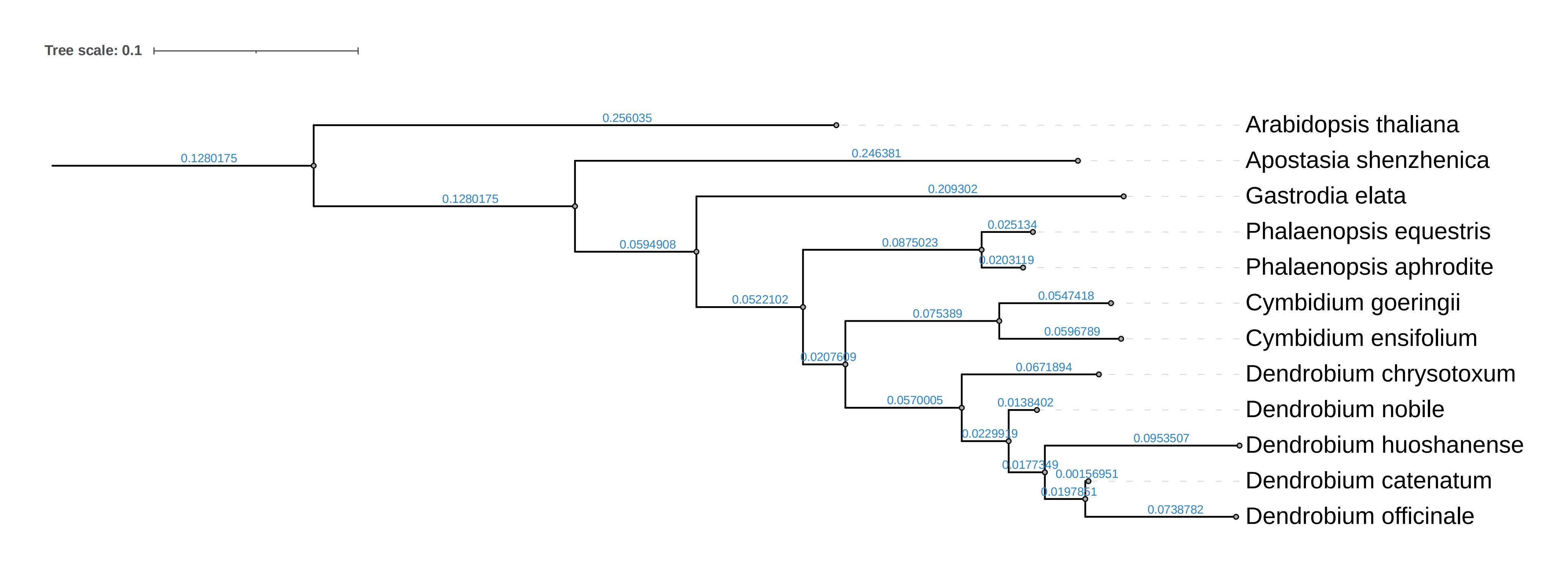

Supplement: Supplementary Figure 1 — The phylogenetic tree of 11 orchid species with publicly available protein sequences based on the identified single-copy genes. A. thaliana was regarded as an outgroup. The tree was visulized by the iTOL online service (https://itol.embl.de/). [file Image_1.jpeg]

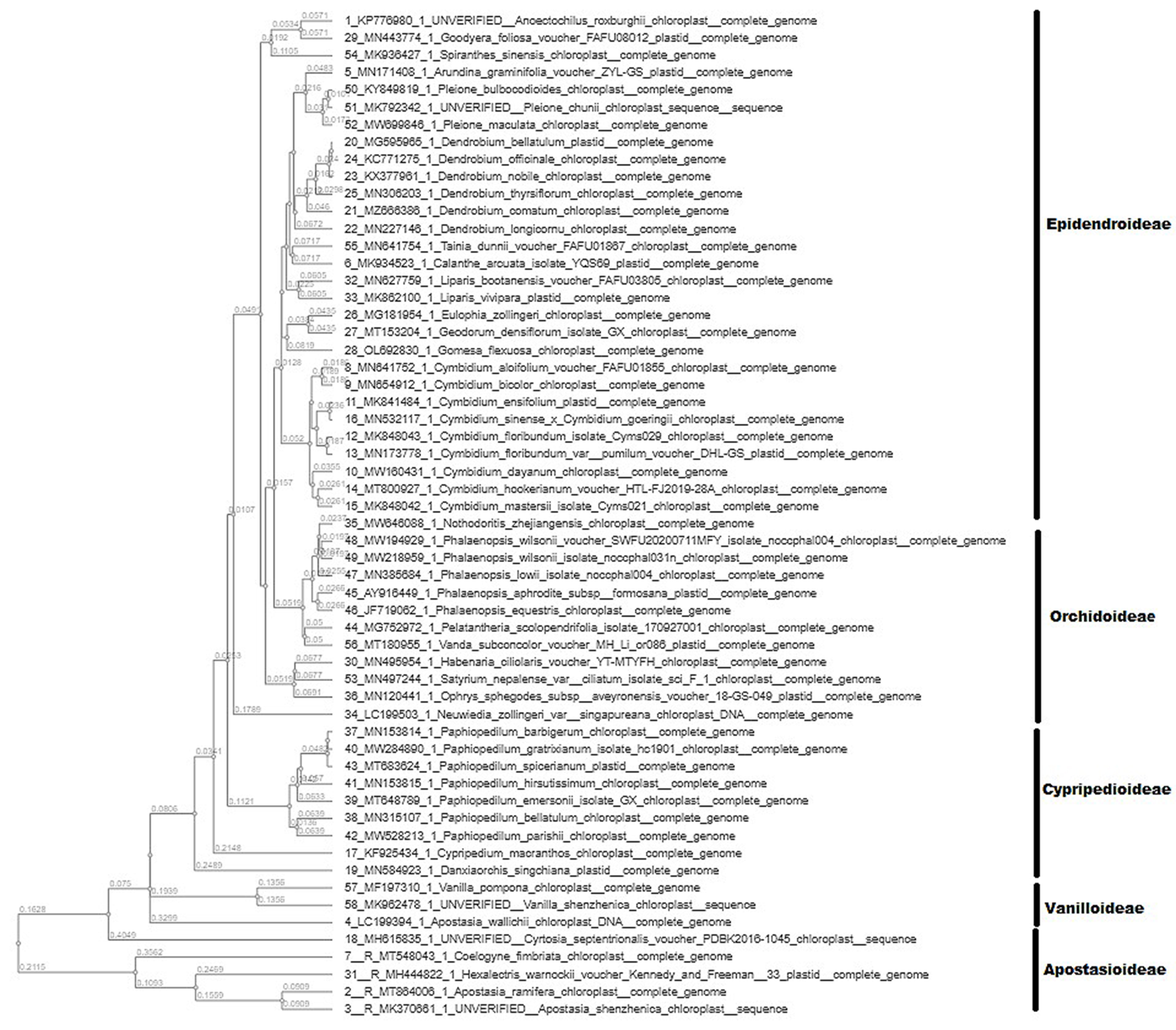

Supplement: Supplementary Figure 2 — The maximum-likelihood (ML) tree of 58 Orchidaceae species based on the chloroplast genomes. Alignments of the cp genomes were performed using MAFFT (v7.505) based on the FFT-NS-2 method (https://mafft.cbrc.jp/alignment/software/). The Archaeopteryx.js tool was used to display the ML tree (https://sites.google.com/site/cmzmasek/home/software/archaeopteryx). [file Image_2.jpeg]
